# Effect of Short-Term Lactic Fermentation on Polyphenol Profile and Antioxidant Capacity in White and Red Quinoa Varieties

**DOI:** 10.3390/foods13152413

**Published:** 2024-07-30

**Authors:** Rui Chu, Eulalia Uaila, Tariq Ismail, Claudia E. Lazarte

**Affiliations:** 1 Division of Food and Pharma, Department of Process and Life Science Engineering, Lunds Tekniska Högskola, Lund University, 22100 Lund, Sweden; ru3300ch-s@student.lu.se (R.C.); eulalia_domingos.uaila@ple.lth.se (E.U.); 2 Department of Chemistry, Science Faculty, Eduardo Mondlane University, Maputo 257, Mozambique; 3 Department of Food Science & Technology, Faculty of Food Science & Nutrition, Bahauddin Zakariya University, Multan 66000, Punjab, Pakistan; tariqismail@bzu.edu.pk

**Keywords:** quinoa varieties, lactic fermentation, bioactive compounds, polyphenols, antioxidant capacity

## Abstract

Quinoa (*Chenopodium quinoa* Willd.) is a pseudocereal originally grown in the Andean region of South America. This study focused on investigating the changes in phenolic profile and antioxidant capacity in white and red quinoa varieties after short-term fermentation with *Lactiplantibacillus plantarum* 299v^®^. During fermentation, pH and lactic acid formation were monitored every three hours until pH was below 4.6. The quinoa phenolic profile was quantified via LC–UV–MS. Total polyphenol content (TPC) and total antioxidant capacity (DPPH and FRAP) were determined via spectrophotometric methods. The findings showed that fermentation resulted in a significant increase (*p* < 0.001) in TPC from 4.68 to 7.78 mgGAE·100 g^−1^ for the white quinoa and from 5.04 to 8.06 mgGAE·100 g^−1^ for the red quinoa variety. Gallic acid was the most abundant phenolic acid detected in unfermented quinoa samples (averaging 229.5 μg·g^−1^). Fermented white quinoa showed an 18-fold increase in epicatechin, while catechin was found only in fermented red quinoa (59.19 μg·g^−1^). Fermentation showed a significantly positive impact on the iron-reducing antioxidant capacity (FRAP) of quinoa (*p* < 0.05). Red quinoa had a higher FRAP antioxidant capacity than the white variety; a similar trend was observed with the DPPH assay. There was a significant correlation (r > 0.9, *p* < 0.05) between TPC and antioxidant capacity. In conclusion, short-time lactic fermentation effectively increased phenolic content and antioxidant capacity in both quinoa varieties. Overall, red quinoa showed higher polyphenol content and antioxidant capacity compared to the white variety.

## 1. Introduction

Quinoa (*Chenopodium quinoa* Willd.) is a pseudocereal originally grown in the Andes region of South America. In recent decades, quinoa has stimulated people’s interest because of its nutritional properties, and it is reported that quinoa could be considered a health-promoting food, with 59.9–74.7% carbohydrate, 13.8–16.5% protein, 2–9.5% fat, and other nutritional compounds such as dietary fiber, vitamin, minerals, and bioactive compounds such as polyphenols [1]. Polyphenols, including phenolic acids, flavonoids, and tannins, have a variety of physiological properties. Among these, phenolic acids include vanillic, syringic, rosmarinic, chlorogenic, and ferulic acid, and flavonoids include quercetin, isoquercetin, kaempferol, and some glycosides [2]. The phenolic compounds in quinoa have been shown to have a good antioxidant capacity, which is the ability to scavenge free radicals and reactive oxygen species [3]. 

Currently, there are almost 250 different varieties of quinoa all over the world, and the identification is based on plant morphology and color of seeds [1]. The most common varieties are white, red, and black quinoa grains [4]. Variety, color, ripeness, and cultivation conditions can all result in differences in polyphenol compounds or other nutrients [2]. It is reported that the content of fatty acids, tocopherols, and carotenoids in quinoa can increase with the darkening of the varieties’ color [5]. Quinoa is a gluten-free crop, and its richness in nutrients and bioactive compounds has been positively associated with the prevention of inflammatory diseases, cancer, allergies, and cardiovascular diseases [6]. On the other hand, it is reported that quinoa contains significant levels of phytates (7.92 to 8.93 g·kg^−1^), which can inhibit the absorption of essential minerals such as iron and zinc [7]. Food processing strategies, such as germination and fermentation, have been successfully used to decrease the levels of phytates in quinoa [8,9].

Food fermentation is a process that can stabilize and alter food components through microbial growth and metabolism. Lactic acid fermentation occurs when lactic acid is produced during the fermentation process. It is widely used in the food industry to preserve foods, improve food safety, and enhance sensory perception. Lactic acid bacteria have a wide range of food applications and are commonly used to ferment dairy products, fish, meat, vegetables, beans, etc. [10]. Our previous studies have shown that fermentation of quinoa contributed to reducing phytate content and consequently increased iron, zinc, and calcium bio-accessibility [9,11]. In those studies, *Lactiplantibacillus plantarum* 299v^®^ (Lp299v) was utilized in quinoa fermentation. Lp299v is a gram-positive lactic acid bacterium that naturally occurs in the human gut as a probiotic strain. Clinical research has demonstrated that this bacterium can help maintain the balance of gut flora, alleviate common intestinal disorders, and increase iron absorption [12].

Previously reported studies on quinoa fermentation showed that quinoa fermentation can improve the total phenol content (TPC) and antioxidant capability; however, these studies involved long-term fermentations of quinoa from 24 h to 5 days [13,14,15]. Long-term fermentation of quinoa and other pseudocereals may be conducive to undesirable side flavors, as shown in our previous research [9,16]. In our consecutive studies, we optimized the fermentation process of quinoa and reduced the fermentation time to 9 h, which allowed a good reduction of phytates while avoiding the formation of undesirable flavors [11,16]. Thereafter, the present study investigates the changes in total phenolic content, phenolic profile, and antioxidant capacities produced by short-time lactic fermentation of red and white quinoa varieties. The findings of this study will improve the understanding of the composition of phenolic compounds in white and red quinoa varieties during short-term fermentations and encourage further research on the development of functional foods and ingredients.

## 2. Materials and Methods

### 2.1. Materials and Chemicals

White quinoa (WQ) and red quinoa (RQ) seeds of the varieties ‘quinoa real blanca’ and ‘pandela’, respectively, are cultivated in the highlands of Bolivia and harvested between April and July. For the present study, the samples were bought from a local market in Cochabamba, Bolivia, in November 2022. Lp299v^®^ (ProbiMage, Lund, Sweden) was used as a starter culture for the quinoa fermentation. Methanol and formic acid were used to extract phenolic compounds in quinoa samples. Folin–Ciocalteu reagent, sodium carbonate, and gallic acid (Sigma-Aldrich, Stockholm, Sweden) were used for total phenol content measurement. Ferric chloride (FeCl_3_·6H_2_O, Fluka, Hanover, Germany), TPTZ (1,3,5-tri(2-pyridyl)-2,4,6-triazine), sodium acetate, DPPH (2,2-diphenyl-1-picrylhydrazyl), and Trolox ((+/−)-6-Hydroxy-2,5,7,8-tetramethylchromane-2-carboxylic acid) (Sigma-Aldrich, Sweden) were used for antioxidant capacity measurement.

### 2.2. Quinoa Fermentation

The quinoa fermentation process was carried out in duplicate, following the procedure previously developed by our research group [11,16]. Briefly, quinoa seeds of different varieties were milled and suspended in distilled water (ratio 1:2 *w*/*v*); Lp299v^®^ (7.35 Log10 CFU·g^−1^ expressed in dry matter (DM)) was inoculated in each suspension and fermented at 37 °C (TS 8136, Termaks, Bergen, Norway) for 9 h. Acidity, quantified as lactic acid content, and pH were monitored every 3 h to control the progress of fermentation. The fermentation was completed when the pH was below 4.6 to ensure the unviability of pathogen or opportunistic bacterium. After fermentation, the samples, fermented white quinoa (FWQ) and fermented red quinoa (FRQ) were freeze-dried (Labconco, Kansas, MO, USA) and stored in vacuum bags for further analysis. The moisture content of each sample was determined gravimetrically following standard procedures according to the Association of Official Analytical Chemists (AOAC) 2000 [17].

### 2.3. pH and Acidity Measurement

pH and acidity values were measured at 0, 3, 6, and 9 h during the quinoa fermentation. According to Nuobariene et al. (2015) [18], pH measurements, in duplicates, were determined in a mixture of 10 g of fermented suspension and 90 mL of distilled water (PHM210 standard pH meter, Radiometer analytical SAS, Lyon, France). Then, 30 mL of the above 100 mL solution was used for the acidity measurement, which was determined through titration with sodium hydroxide. For the calculation, the equivalence of 1.0 mL of 0.1 N sodium hydroxide was equivalent to 9.0 × 10^−3^ g lactic acid [18]. The results were expressed as g·kg^−1^ sample DM of lactic acid.

### 2.4. Extraction of Polyphenols

Unfermented and fermented samples of white and red quinoa varieties were extracted following the procedure described by Rocchetti et al. (2019) [13]. Briefly, 2 g of each sample, in duplicate, were extracted in 25 mL of 80% methanol acidified with 0.1% formic acid solution. Samples were homogenized for 2 min at 16,000 rpm using an Ultra-Turrax (Ystral X10/25, Ballrechten-Dottingen, Germany), shaken for 1 h at 1000 rpm, and then centrifuged for 10 min at 2000 rpm, 4 °C (Allegra X-15 R Centrifuge, Beckman Coulter, Brea, CA, USA). The final extracts were obtained by filtering the supernatant through 0.2 μm PTFE syringe filters and stored at −18 °C for further analysis.

### 2.5. Total Polyphenol Content (TPC)

TPC was measured using the method of Singelton and Rossi (1965) [19]. Briefly, 0.5 mL of methanolic extract was mixed with 0.625 mL of diluted Folin–Ciocalteu reagent (ratio 1:9, diluted in distilled water) and 0.5 mL of 7.5% sodium carbonate. Distilled water was added to a total volume of 10 mL. The samples were vortexed immediately and incubated in the dark for 60 min at room temperature. The experimental wavelength was determined through wavelength scanning to obtain maximum absorbance, which resulted in 636 nm (Varian Cary 50 Bio UV–Visible Spectrophotometer, Varian, CA, USA). Gallic acid was utilized for a standard curve with the following points: 0, 10, 25, 40, 55, 70, 85, and 100 mg·L^−1^. The results were expressed in mg gallic acid equivalent per gram dry-weight basis (mgGAE·g^−1^ dwb).

### 2.6. Antioxidant Capacity 

#### 2.6.1. Antioxidant Capacity via Ferric Reducing Antioxidant Power Assay (FRAP)

The measurement was performed according to Rocchetti et al. (2019) [13]. The FRAP working solution was freshly prepared each time of analysis by mixing 300 mM acetate buffer, 20 mM ferric chloride solution, and TPTZ (2,4,6-tri(2-pyridyl)-1,3,5-triazine) solution. Subsequently, 2.7 mL of FRAP solution was mixed with 0.3 mL of a five-fold dilution of the methanolic grain extracts and then incubated at 37 °C in a water bath for 40 min. The absorbance was measured at 594 nm (Varian Cary 50 Bio UV–Visible Spectrophotometer, Temecula, CA, USA).

#### 2.6.2. Antioxidant Capacity and Inhibition Ratio via Scavenging DPPH

The DPPH assay was conducted according to the method described by Goupy et al. (1999) [20]. Briefly, 0.5 mL of pure methanolic extract and 2.25 mL of DPPH working solution were mixed and incubated in the dark at room temperature for 30 min before the absorbance was measured at 517 nm (Varian Cary 50 Bio UV–Visible Spectrophotometer, CA, USA). Trolox solution was used as a standard for the calibration curve. Results are presented in mg Trolox equivalent per 100 g dry-weight basis (mgTE·100 g^−1^ dwb). Simultaneously, 80% methanol was used as the control sample to calculate the inhibition ratio (%) using Equation (1):

Inhibition Ratio (%) = IR% = (A_1_ − A_2_) × 100/A_1_, (1)

A_1_: absorbance of control methanol;

A_2_: absorbance of testing samples.

The testing sample concentration was referred to as IC50 when the inhibition ratio was 50% [21]. To calculate the IC_50_ value, samples were diluted with distilled water at a proportion of 1:1 and 1:3 before being mixed with DPPH. The value was determined using two points enclosing the 50% inhibition ratio. The final antioxidant capacity was expressed using Trolox Equivalent Antioxidant Capacity (TEAC) values [22]. Trolox was used to achieve the IC_50_ value, with serial dilutions of 10, 20, 30, 40, 50, and 60 mg·L^−1^.

TEAC = (IC_50_Trolox/IC_50_Sample) × 10^5^. (2)

### 2.7. Polyphenol Profile via Liquid Chromatography LC–UV–MS

Identification and quantification of the phenolic profile in the extracts were conducted according to Plaza M. et al. (2013) [23] with some modifications. The extracted samples were concentrated using a rotavapor (Heidolph Rotary Evaporator, Schwabach, Germany) and reconstituted with 2 mL of 80% methanol. The extracts were filtered through a 0.2 μm PTFE membrane and collected in vials for analysis. UPLC–MS analysis was carried out using UPLC systems (Waters Acquity UPLC and Waters XEVO-G2, Milford, MA, USA). The conditions for MS detection were as follows: 3.0 kV in ESI+ and −2.5 kV in ESI-; cone voltage: +30 V/−30 V; source temperature: 120 °C; desolvation temperature: 500 °C; cone gas: 50 L/h; desolvation gas flow: 800 L/h; acquisition: full scan 100–1200 *m*/*z*. Chromatographic separations were conducted using a C18 column (2.1 mm × 100 mm) (Waters Acquity HSS T3, MA, USA). Mobile phase A (MPA) was 0.1% formic acid, and mobile phase B (MPB) was acetonitrile containing 0.1% formic acid. The UV detector was configured to record absorbance in the 200–500 nm range. Identification of polyphenol compounds was based on retention times, which were determined from a standard mixture using both UV and MS data. Confirmation of co-eluting peaks was achieved via the analysis of single standards. Results are expressed as μg per gram dry-weight basis.

### 2.8. Statistical Analysis

The fermentation process was carried out in duplicate, as was the analysis of the different parameters. Results are presented as mean values with standard deviations. An unpaired *t*-test was computed using GraphPad Prism 9 (GraphPad Software, Boston, MA, USA) to analyze significant differences among processes and quinoa varieties for all parameters and changes in polyphenol profile due to fermentation. Normality tests and nonparametric Spearman tests were performed to obtain correlation coefficients between total phenol content and antioxidant capacity. The level of significance was reported at *p* < 0.05.

## 3. Results

### 3.1. pH and Lactic Acid Production during the Quinoa Fermentation

**Figure 1:**
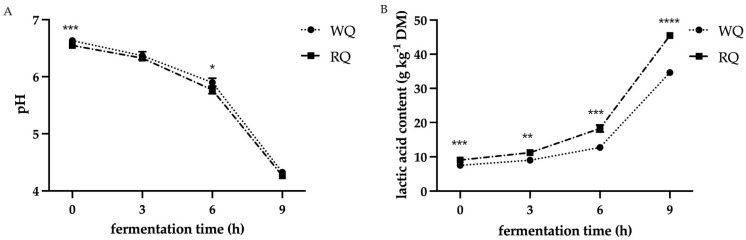
Changes in pH and lactic acid content during lactic fermentation of white and red quinoa varieties: (**A**) pH, (**B**) lactic acid content. Significant differences are shown using * *p* < 0.05, ** *p* < 0.01, *** *p* < 0.001, and **** *p* < 0.0001 levels. WQ: white quinoa, RQ: red quinoa.

The results of pH and lactic acid production during the fermentation of white and red quinoa varieties are shown in Figure 1. It was found that pH decreased as fermentation time increased. Conversely, lactic acid content increased with fermentation time. Before fermentation, WQ had a pH value of 6.63 ± 0.02, while RQ had a pH of 6.55 ± 0.01. During fermentation, the pH difference between the white and red quinoa varieties was not significant. In the first 6 h of fermentation, lactic acid produced during fermentation showed a slow increase from 7.53 ± 0.42 g·kg^−1^ and 9.05 ± 0.02 g·kg^−1^ to 12.71 ± 0.86 g·kg^−1^ and 18.26 ± 1.04 g·kg^−1^ for white and red quinoa varieties, respectively. Subsequently, rapid changes occurred between 6 and 9 h, and by the end of fermentation, red quinoa (45.50 ± 0.79 g·kg^−1^) produced considerably more lactic acid than white quinoa (34.66 ± 0.75 g·kg^−1^). It is worth mentioning that lactic acid content in RQ was consistently higher than in WQ, even in the unfermented samples. The final pH measurements for FWQ and FRQ were not significantly different, with values of 4.33 ± 0.02 and 4.27 ± 0.06, respectively.

### 3.2. Total Phenol Content of White and Red Quinoa Varieties before and after Fermentation

The results of TPC are expressed as gallic acid equivalent (Table 1). The results indicate that short-term lactic acid fermentation significantly (*p* < 0.05) enhanced the total phenol content in white and red quinoa varieties. Red quinoa contained significantly higher TPC than white quinoa. After fermentation, TPC in white quinoa increased by 66%, from 4.68 ± 0.05 to 7.78 ± 0.07 mg GAE·g^−1^, while the increase in red quinoa was slightly less (60%), with values rising from 5.04 ± 0.03 to 8.52 ± 0.27 mg GAE·g^−1^. Nonetheless, there was no significant difference between FWQ and FRQ in terms of TPC. 

### 3.3. Antioxidant Capacity of White and Red Quinoa Varieties before and after Fermentation

A similar trend of results was found for antioxidant capacity as measured using the FRAP assay. FRAP results for unfermented samples showed that RQ had a higher antioxidant capacity than WQ. Fermentation showed a significantly positive impact on the iron-reducing antioxidant capacity of quinoa (*p* < 0.05). The values for FWQ and FRQ increased from 4.83 ± 0.20 and 6.42 ± 0.25 (mg TE·g^−1^) to 6.91 ± 0.13 and 8.52 ± 0.27 (mg TE·g^−1^), respectively. FRAP showed increases of 43% for FWQ and 33% for FRQ. DPPH scavenging results are presented in terms of antioxidant capacity as Trolox Equivalent and Equivalent Antioxidant Capacity values (TEAC). These results followed the same trend as those found for FRAP, but they were 6 to 11-fold higher. After lactic acid fermentation, the DPPH antioxidant capacity for FWQ and FRQ showed an increase from 48.93 ± 0.21 to 57.61 ± 0.58 (mg TE·100 g^−1^) and from 54.43 ± 0.39 to 64.62 ± 0.57 (mgTE·100 g^−1^), respectively. Additionally, there were significant differences between fermented and unfermented groups, as well as between white and red quinoa (*p* < 0.05). In terms of TEAC, the results for WQ and RQ were 36.86 ± 0.52 and 43.31 ± 0.29, respectively. Fermentation led to a visible increase in antioxidant capacity for both quinoa varieties. It was demonstrated that TEAC values for WQ and RQ varieties increased nearly one-fold after fermentation (Table 1). Furthermore, the values presented in Table 1 indicate that there was a strong correlation between TPC and TAC as measured using both methods, FRAP and DPPH.

### 3.4. Polyphenol Profile in Unfermented and Fermented Samples 

Table 2 shows the polyphenol composition of white and red quinoa varieties before and after fermentation. A total of sixteen phenolic compounds were detected in the quinoa samples, of which the predominant ones were gallic acid in WQ at 260.12 ± 14.17 μg·g^−1^ and in RQ at 198.83 ± 9.56 μg·g^−1^, while epicatechin was predominant in FWQ at 297.03 ± 23.67 μg·g^−1^. Interestingly, catechin was only detected in the FRQ samples (59.19 ± 17.86 μg·g^−1^). The concentrations of chlorogenic acid significantly increased by 139.96% and 439.58% after the fermentation of white and red quinoa, respectively. Moreover, the concentrations of the following polyphenols in WQ significantly increased after fermentation: 4-hydroxybenzoic acid (127.25%), vanillic acid (100.20%), epi-catechin (1738%), syringic acid (68.25%), quercetin (167.86%), and caffeic acid (51.43%). The concentrations of t-cinnamic acid, kaempferol, ferulic acid, p-coumaric acid, caffeic acid, and resveratrol were found in concentrations below 5 μg·g^−1^. Vanillin was only found in RQ, and p-coumaric acid was only detected in WQ. t-Cinnamic acid was only found in unfermented quinoa grains, while kaempferol was only detected in fermented quinoa grains. 

**Figure 2:**
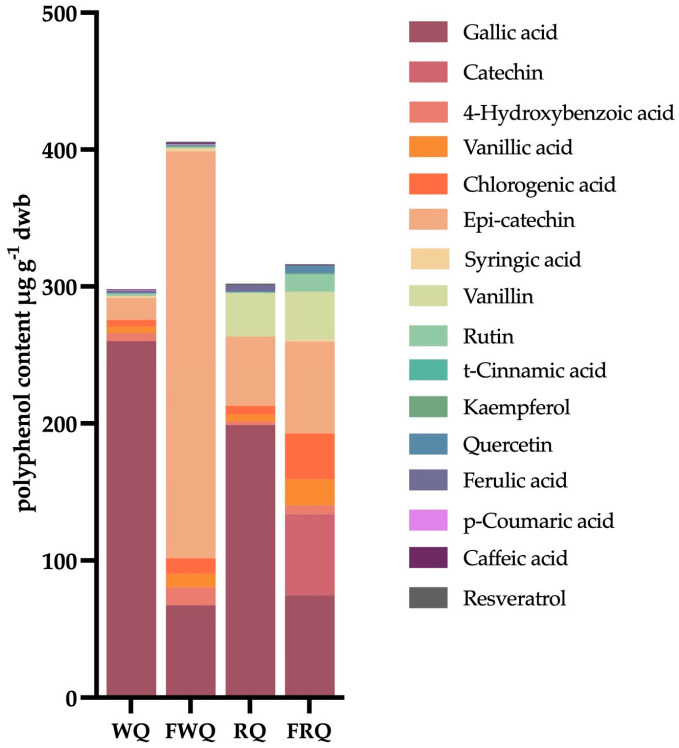
Changes in the polyphenol profile of each quinoa variety before and after fermentation. Results are displayed based on mean values. WQ: unfermented white quinoa; RQ: unfermented red quinoa; FWQ: fermented white quinoa; FRQ: fermented red quinoa.

**Figure 3:**
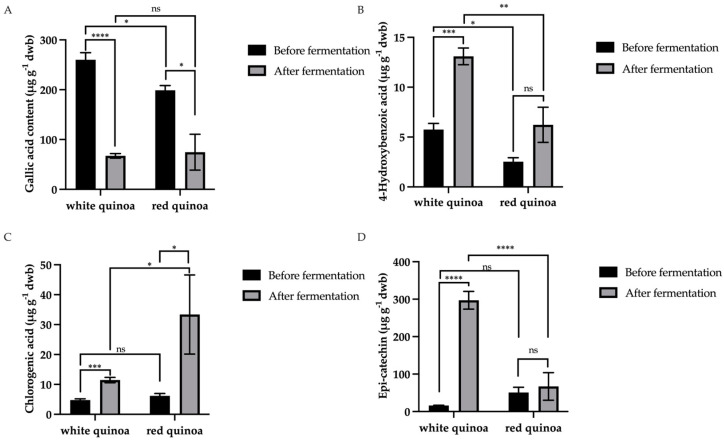
Most significant changes in the concentration of selected polyphenols in quinoa after short-term lactic fermentation: (**A**) gallic acid, (**B**) 4-hydroxybenzoic acid, (**C**) chlorogenic acid, and (**D**) epicatechin in white and red quinoa varieties before and after fermentation. Significant differences are shown using * *p* < 0.05, ** *p* < 0.01, *** *p* < 0.001, and **** *p* < 0.0001 levels. ns: no significant different.

Figure 2 shows how the polyphenol profile changed due to short-term lactic fermentation. The highest percentage of phenolic compounds detected in each quinoa sample was gallic acid in WQ and RQ, epicatechin in FWQ, and catechin, which was detected only in FRQ. Gallic acid concentration dropped considerably after fermentation in both quinoa types (*p* < 0.001). Before fermentation, WQ contained more gallic acid and 4-hydroxybenzoic acid compared to RQ. Furthermore, FWQ had higher levels of 4-hydroxybenzoic acid, epi-catechin, syringic acid, and caffeic acid compared to FRQ. Figure 3 shows that WQ had a substantial rise in 4-hydroxybenzoic acid, chlorogenic acid, and epicatechin after fermentation (*p* < 0.001), whereas RQ had a significant increase in catechin, chlorogenic acid, rutin, and quercetin.

Examples of chromatograms for standards and samples before and after fermentation are presented in Supplementary Material; Figure S1: Chromatogram of mixed polyphenol standards and Figure S2: Example of a chromatogram of individual polyphenol standards. Figures S3–S6 correspond to examples of chromatograms of phenolic compounds in unfermented and fermented white and red quinoa varieties.

## 4. Discussion

The main findings of this research show that short-term lactic fermentation of quinoa resulted in an increase in TPC by about 60% in both white and red quinoa varieties. Fermentation showed a significant positive impact on the iron-reducing antioxidant capacity of quinoa (*p* < 0.05) as well as on the DPPH antioxidant capacity. Sixteen phenolic compounds were detected and quantified in quinoa samples before and after fermentation, and significant changes were found in how the phenolic profile was affected by lactic fermentation. 

### 4.1. Effect of Lactic Acid Fermentation on pH and Acidity

The findings of the current study showed that short-term lactic acid fermentation of quinoa proceeded with a decrease in pH and the formation of lactic acid. Similar findings were reported in previous studies on the lactic fermentation of quinoa using *Lactiplantibacillus plantarum* 299v [9,11,16]. Figure 1 shows that the pH of both white and red quinoa dropped steadily throughout the first 6 h and drastically decreased between 6 and 9 h. It has been claimed that the pH drop during quinoa fermentation could be influenced by the production of organic acids and the activity of endogenous microorganisms [24]. The decrease in pH was steady in the first hours and accelerated later, as reported in previous studies, which noted that it takes some time for microbial activity to adapt to the fermentation environment [25]. Consequently, endogenous microorganisms accelerated the degradation of carbohydrates into organic acids in the later stage of fermentation, leading to a more pronounced pH decrease during the last 3 h of fermentation. 

An interesting finding of Ayub et al. (2021) [11] was that the most significant reduction in pH during quinoa fermentation occurred between 3 and 6 h, which happened earlier than in the current study. Comparing the two experimental conditions, quinoa was also fermented for 9 h at 37 °C with the same starter culture in their study. The difference was that in their study, roasted quinoa was used; roasting may have produced some lactic acid, and that could promote a faster pH reduction [11]. Thus, the final production of lactic acid in their study was 90.9 g·kg^−1^, which was significantly higher than the result in the current study (34.66 g·kg^−1^ for FWQ and 45.50 g·kg^−1^ for FRQ). However, it is worth noting that the lactic acid content (21.6 g·kg^−1^) of unfermented quinoa in the Ayub et al. (2021) [11] study was initially higher than the results in this paper (12.71 g·kg^−1^ for FWQ and 18.26 g·kg^−1^ FRQ). The quinoa used in that study was from Bolivia but bought in Sweden. In contrast, Castro-Alba et al. (2019) [9] found that the lactic acid content of quinoa flour varied roughly around 10 g·kg^−1^, and the pH was around 6.2 before fermentation, which was more in line with the findings of this study. They also used quinoa from Bolivia. Variations in pH and lactic acid content in unfermented quinoa grains may be due to differences in quinoa varieties, cultivars, and environmental factors [26].

There was a significant difference in lactic acid production during the fermentation of white and red quinoa varieties (Figure 1). Before fermentation, red quinoa contained slightly higher initial lactic acid content than the white variety. As fermentation continued, red quinoa produced nearly 10 g·kg^−1^ more lactic acid than the white variety by the end of fermentation. This suggested that there are variations among quinoa species. Research conducted by Song et al. (2021) [1] showed that different colored quinoas (white, red, and black) had varying amounts of total organic acids, with red quinoa holding the highest concentration of lactic acid among the varieties. 

### 4.2. TPC and Phenolic Profile of White and Red Quinoa Varieties before and after Fermentation

Overall, the current study illustrated that short-term lactic acid fermentation played an important role in increasing TPC. Fermentation enhanced TPC values by more than 50% in white and red quinoa varieties. Red quinoa grains contained significantly higher TPC than the white variety. These results are in concordance with previous studies where it was shown that quinoa with darker colors, such as red or black varieties, contained higher TPC compared to the white variety [5,26,27]. In Han et al. (2019) [28], a study about the characterization of phenolic compounds of colored quinoas, they reported that quinoa with dark colors (red and black) contained a higher concentration of betalains. The phenolic hydroxyl group in betalains can react actively with the Folin–Ciocalteu reagent, leading to an increase in TPC. Also, the amount of betalains was positively correlated with TPC values [28]. For this study, black quinoa was not included as it was not available at the time of sample collection. However, it would be of great interest to include black quinoa as well as other varieties in further studies. It is worth mentioning that different cultivars and growing locations play an important role in TPC values. 

In this study, it was shown the positive effect of short-term lactic fermentation on increasing the level of TPC and antioxidant capacities. Other authors have also stated the positive effect of quinoa fermentation on increasing TPC [13,14,29]. However, those results were obtained from long-term fermentations, which may alter the sensory characteristics of quinoa. For example, Rocchetti et al. (2019) [13] reported that the fermentation of quinoa with *Pediococcus pentosaceus* at 30 °C for 24 h increased TPC from 59.6 mgGAE·100 g^−1^ to 70.9 mgGAE·100 g^−1^. It has been reported that phenolic substances can be transferred from a bound form to a free state during fermentation, a transformation attributed to bond breakdown, enzyme activities, and microbial metabolism [30]. Additionally, fermentation can trigger cereal cell wall structural breakdown, resulting in the liberation and/or production of a variety of bioactive chemicals such as polyphenols [30,31]. Sánchez-García et al. (2022) reported results of the solid-state fermentation of quinoa seeds using *Pleurotus ostreatus* at 28 °C for 14 days. In this process, TPC changed slowly and did not vary significantly during the fermentation period. The results showed that total phenol content increased from 1.57 mgGAE·g^−1^ (day 0) to 2.5 mgGAE·g^−1^ (day 8), while it decreased to around 1.6 mgGAE·g^−1^ by the end of fermentation [32]. It is speculated that the differences in the impact of fermentation on the TPC of quinoa may be related to the starter culture used and the fermentation technique [33]. 

Regarding the phenolic profile, gallic acid was the main phenolic acid detected and accounted for the highest proportion in the unfermented white and red quinoa samples in this study. This finding is comparable to values reported by Han et al. (2019) [28], who analyzed three white quinoa varieties, two red quinoa varieties, and two black quinoa varieties. Among them, each color had one variety from Peru, while the others came from the local market in China. The result indicated that gallic acid was the main phenolic compound in quinoa grains, ranging from 167.79 μg·g^−1^ to 331.37 μg·g^−1^ [28], which is in line with those found in this study. While the gallic acid detected in Pellegrini et al. (2018) [34] was relatively low, their research included five varieties of white and red quinoas from Bolivia, Spain, and Peru, with gallic acid concentrations in dwb ranging from 72.19 μg·g^−1^ to 95.86 μg·g^−1^. Their results revealed that polyphenol profile varies significantly between different quinoa varieties. White quinoa from Peru had the greatest gallic acid level (95.86 μg·g^−1^), even exceeding that of red Bolivian quinoa (90.41 μg·g^−1^), while one of the Bolivian white quinoa samples had the lowest content (72.19 μg·g^−1^). Apart from gallic acid, the content of 4-hydroxybenzoic acid (106.33–141.09 μg·g^−1^) and vanillic acid (91.94–108.96 μg·g^−1^) in their study was also considerably high. Additionally, some other phenolics were discovered in their research, such as neohesperidin (41.79–112.88 μg·g^−1^) and hesperidin (28.59–44.05 μg·g^−1^) [34]. Generally, it is challenging to compare results with previous research. It has been reported that the diversity of phenolic compounds in quinoa is not only related to the variety and species but also depends on agricultural conditions, environmental factors, and post-harvest treatments, as well as extraction methods used during the analysis [35].

Epicatechin content was the highest flavonoid detected in quinoa, especially in FWQ. In contrast, catechin was only detected in FRQ. Melini et al. (2021) [30] concluded that catechin was detected only in black and red quinoas (in the bound form). In their study, the catechin content in red quinoa increased from 0.055 to 0.130 mg·100 g^−1^ after fermentation with *R. oligosporus* at 31 °C for 4 days [30]. As shown in Figure 3, there was a very significant decrease in gallic acid content after fermentation (FWQ: 3.86-fold decrease; FRQ: 2.66-fold decrease), while the content of 4-hydroxybenzoic acid increased 2.27-fold in FWQ and 2.46-fold in FRQ. Vanillic acid increased 2-fold in FWQ, and chlorogenic acid increased 2.4-fold in FWQ and 5.4-fold in FRQ. Carciochi et al. (2016) [14] reported that p-OH benzoic acid and vanillic acid were not detected after natural fermentation, and ferulic acid showed a 7.14-fold decrease. However, in their study, fermentation with *S. cerevisiae* led to increases of 8.31-fold, 1.42-fold, and 2.35-fold for OH benzoic acid, vanillic acid, and ferulic acid, respectively. It is reported that phenolic compounds may be degraded during the fermentation process, and it is related to the microorganism species involved. The increased activity of phenolic reductase, phenolic decarboxylase, and glucosidase may give rise to the metabolism of certain phenolic acids and flavonoid glucosides during fermentation [14]. 

A plethora of literature is available on the health significance of non-nutrient bioactive compounds identified in our study. Epicatechin was found to be one of the high-recovery bioactive compounds in fermented quinoa. It exhibits antioxidant and anti-inflammatory properties and prevents/improves health conditions such as diabetes and cardiovascular and cerebrovascular diseases [36]. Epicatechin can cross the blood–brain barrier and modify the rheological properties of blood and is hence considered beneficial for neuropsychological and cardiovascular health [37]. Catechin is one of the strong antioxidants of plant origin, and it was found in higher concentrations in fermented red quinoa. It has also been reported as one of the safest polyphenols bearing plausible antimicrobial, antiviral, anti-allergic, anti-inflammatory, and anticancer properties [38]. Fermented white and red quinoa varieties were also found to be carriers of vanillin, rutin, kaempferol, and chlorogenic acid, all of which are dietary phenolic acids significant for human health. 

### 4.3. Antioxidant Capacity of White and Red Quinoa Varieties before and after Fermentation 

DPPH and FRAP assays were chosen to determine the antioxidant capacity of quinoa and fermented quinoa samples. These measurements differ in various aspects, such as their chemistry, the generation of different radicals, and the way they target certain molecules. Given that various antioxidant compounds may act through different mechanisms, it is recommended to use a combination of methods, as no single method can completely evaluate the TAC of foods.

The TAC results found in WQ were comparable to those reported in Kaur et al. (2016) [39], where they used white quinoa produced locally in India, with values of 59.6 mgTE·100 g^−1^ and TEAC of 37.3 mgTE·100 g^−1^ analyzed using the DPPH method. On the other hand, the FRAP results for WQ in our study (4.83 mgTE·g^−1^) was more than five times higher than that reported for Indian quinoa seeds (84.46 mgTE·100 g^−1^) [39]. The results of our study are in line with those reported by Alvarez-Jubete et al. (2010) [21], where quinoa grains also from Bolivian origin (no color clarification) exhibited a TEAC value of 34.8 mgTE·100 g^−1^. This was similar to the TEAC value for WQ in our study (36.86 mgTE·100 g^−1^) and around 20% lower than the TEAC result for RQ (43.31 mgTE·100 g^−1^). 

Pellegrini et al. (2018) [34] reported FRAP values for five white and red quinoa varieties from Bolivia, Spain, and Peru, and their results ranged from 2.53 to 4.88 mgTE·g^−1^ (dwb), which were comparable to those in this study. However, their DPPH results were between 2.07 and 5.35 mgTE·g^−1^ [34], which were around 4 to 10 times higher than the results in this study. It is important to note that analytical methods in different studies may differ, and factors such as solvent type, reaction time, and final expression of results can all lead to variations. Inglett et al. (2015) [40] detected the antioxidant capacity of quinoa using different extraction solvents (water, 50% ethanol, and absolute alcohol) and concluded that the antioxidant activity of phenolic compounds in quinoa extracts with the first two solvents increased by 30–50% [40]. Furthermore, the trend we found between quinoa’s color and antioxidant capacity agrees with the findings presented by Pedrali et al. (2023) [26], with a higher antioxidant capacity in red quinoa seeds. The argument was that red quinoa contains more carotenoids and twice as many γ-tocopherols as white quinoa, and the antioxidant capacity was likely positively correlated with the content of these compounds [5].

The findings in this study showed that short-term lactic fermentation could effectively increase the antioxidant capacity. Many previous studies have confirmed the positive effect of fermentation on increasing antioxidant capacity, although in long-term fermentation [14,30,31,41]. Carciochi et al. (2016) [14] studied the effect of fermentation with *Saccharomyces cerevisiae* for 24 h at 30 °C on quinoa seeds from Argentina. Their results showed an increase of about 43% for DPPH and 51% for FRAP after fermentation [14]. Compared to the results of the current study, the increase in FRAP was similar, while the increase in DPPH was slightly lower in our study. Li et al. (2018) [29] indicated that half-inhibition concentration values for scavenging DPPH of fermented quinoa were 50% lower than those of unfermented quinoa. They attributed the increased antioxidant properties to the higher release of free phenols during fermentation, and some amino acids, such as histidine, tyrosine, and methionine, have been reported to have free radical scavenging activity [29]. Remarkably, in Rocchetti et al. (2019) [13], FRAP in raw quinoa was 15.4 μmolGAE·100 g^−1^ DW, while there was no ferric-reducing antioxidant power detected after fermentation with *P. pentosaceus* and/or *L. paracasei* at 30 °C for 24 h [13]. Thus, published results indicate that changes in antioxidant capacity due to fermentation are greatly influenced by fermentation conditions and the starter cultures used. 

The current study showed a significant correlation between TPC and antioxidant capacity (DPPH and FRAP). Due to the non-normal distribution of TPC data, Spearman correlation coefficients were calculated to analyze the result. The correlation between TPC and FRAP was 0.958, and the correlation between TPC and DPPH was 0.902. Likewise, Alvarez-Jubete et al. (2010) reported a very strong correlation (R^2^ = 0.99) for both TPC vs. DPPH and TPC vs. FRAP in quinoa extracts [21]. On the other hand, a study by Nsimba et al. (2008) [41] found weak correlations in quinoa extracts: TPC vs. DPPH (R^2^ = 0.2049) and TPC vs. FRAP (R^2^ = 0.4833). In their study, they had two quinoa varieties from Japan and Bolivia. They hypothesized that this discrepancy could be because the antioxidants in quinoa seeds are non-phenolic [41]. Moreover, Pedrali et al. (2023) [26] reported that the correlation between total phenol content and antioxidant properties (FRAP) might be related to the quinoa variety. Their study utilized five quinoa cultivars (Salcedo Inia, Kancolla, Chullpi Real, Pasankalla, and Negra Collana) to explore the effect of variety on antioxidant profile. The findings showed that there was no correlation between TPC and FRAP in the Chullpi and Kancolla cultivars [26]. In general, the correlation between TPC and antioxidant capacity is complex. Li et al. (2021) argued that, in addition to phenols, other substances present in food, such as carotenoids, sterols, ascorbic acid, and vitamin E, can also exhibit antioxidant properties. Meanwhile, the methods used for determining antioxidant capacity using different analytical principles probably resulted in various discrepancies. In addition, some bioactive compounds may not show antioxidant properties [27]. It is pointed out that reaction conditions such as antioxidant solubility, oxidation state, pH, and substrate type could also influence the results [21]. 

Fermentation of seed grains is claimed to improve nutritional and health-promoting properties like antioxidant activities. Data suggest that fermentation facilitates the bioconversion of bound phenolics from conjugated forms to free forms through the activity of certain enzymes or the metabolic activity of fermenting microflora. Free forms of phenolics and their free aglycones have the potential to deliver higher antioxidant activity than their counterparts [42]. In line with the findings of our study, Jiang et al. (2024) reported that fermentation effectively enriched the bioactive compound profile of foods and enhanced biological activity [43]. A similar effect was documented by Liu et al. (2017) in their study on lactic acid-fermented rice bran, where researchers reported increased antioxidant activity as a result of higher amounts of free and soluble conjugated phenolic compounds in fermented bran [44]. The superiority of bioactive compounds regarding their antioxidant activity and effectiveness in different systems, such as aqueous or lipid systems, is also associated with their molecular weight. Monomers, dimers, and trimers are superior antioxidants in aqueous phases, whereas high molecular weight procyanidins are more effective antioxidants in lipid systems, as found by Lotito et al. (2000) [45]. Our study identified fermented quinoa as a better carrier of available, low molecular weight polyphenols and procyanidins, such as resveratrol, catechin, and epicatechin, which may be related to the higher antioxidant activity of fermented quinoa seed extracts compared to non-fermented ones. However, it is of great importance to conduct follow-up studies investigating the quality, functionality, and storage stability of fermented quinoa flour.

## 5. Conclusions

In conclusion, controlled short-time lactic fermentation of quinoa with Lp299v^®^ played an important role in increasing total polyphenol content and antioxidant capacity in both white and red quinoa varieties. Moreover, this study found a strong correlation between TPC and antioxidant capacity (DPPH and FRAP). In the analysis of phenolic compounds, gallic acid was the most abundant phenolic acid detected in unfermented quinoa grains, while fermented white and red quinoas showed the highest levels of epicatechin and catechin, respectively. Epicatechins and catechins are reported to exhibit higher antioxidant capacities than gallic acid, aligning with the significant increase in antioxidant capacity in the fermented samples. Furthermore, polyphenols with reported positive health effects, such as quercetin, kaempferol, and 4-hydroxybenzoic acid, were significantly increased after the fermentation process. 

Thus, short-term lactic fermentation of quinoa may offer an excellent strategy to produce polyphenol-enriched products and functional foods. This study fosters further research into the nutritional and health effects of fermented quinoa grains through in vitro and in vivo studies. 

## Figures and Tables

**Table 1:** Effect of lactic fermentation on TPC, antioxidant capacity via FRAP, and DPPH savenging activity. Results are presented as mean ± Sd on a dry weight basis (dwb).

			TAC		
	Moisture Content %	TPC mg GAE·g^−1^	FRAP Assay mg TE·g^−1^	DPPH Scavenging Capacity	
				TEAC	mgTE·100 g^−1^
WQ	11.18 ± 0.20 ^a^	4.68 ± 0.05 ^Aa^	4.83 ± 0.02 ^Aa^	36.86 ± 0.52 ^Aa^	48.93 ± 0.21 ^Aa^
FWQ	-	7.78 ± 0.07 ^Ba^	6.91 ± 0.13 ^Ba^	79.04 ± 2.40 ^Ba^	57.61 ± 0.58 ^Ba^
RQ	11.27 ± 0.37 ^a^	5.04 ± 0.30 ^Aa^	6.42 ± 0.25 ^Ab^	43.31 ± 0.29 ^Ab^	54.43 ± 0.39 ^Ab^
FRQ	-	8.06 ± 0.14 ^Bb^	8.52 ± 0.27 ^Bb^	95.57 ± 1.89 ^Bb^	64.62 ± 0.57 ^Bb^
Correlation					
TPC vs. FRAP			r = 0.958		
Correlation					
TPC vs. DPPH			r = 0.902		

In the same column, superscript lowercase letters indicate differences between quinoa varieties and processing methods. Superscript uppercase letters indicate differences between unfermented and fermented samples of the same variety. Significant differences were computed at *p* < 0.05. WQ: white quinoa unfermented; RQ: red quinoa unfermented; FWQ: fermented white quinoa; FRQ: fermented red quinoa; TPC: total phenol content. TAC, total antioxidant capacity. TEAC, equivalent TAC. FWQ and FRQ were freeze-dried samples with moisture content below 0.5%.

**Table 2:** Polyphenol profile of white and red quinoa before and after fermentation.

Phenolic Compounds	Content (μg·g^−1^ dwb)			
	WQ	FWQ	RQ	FRQ
Gallic acid	260.12 ± 14.17 ^Bb^	67.31 ± 4.62 ^Aa^	198.83 ± 9.56 ^Ba^	74.67 ± 36.10 ^Aa^
Catechin	ND	ND	ND	59.19 ± 17.86
4-Hydroxybenzoic acid	5.76 ± 0.61 ^Ab^	13.09 ± 0.84 ^Bb^	2.53 ± 0.40 ^Aa^	6.23 ± 1.76 ^Aa^
Vanillic acid	4.92 ± 0.60 ^Aa^	9.85 ± 0.64 ^Ba^	5.20 ± 0.60 ^Aa^	19.21 ± 15.61 ^Aa^
Chlorogenic acid	4.78 ± 0.41 ^Aa^	11.47 ± 0.90 ^Ba^	6.19 ± 0.80 ^Aa^	33.40 ± 13.23 ^Bb^
Epi-catechin	16.16 ± 0.62 ^Aa^	297.03 ± 23.67 ^Bb^	50.76 ± 14.12 ^Aa^	67.01 ± 36.79 ^Aa^
Syringic acid	1.26 ± 0.20 ^A^	2.12 ± 0.25 ^Bb^	ND	1.12 ± 0.18 ^a^
Vanillin	ND	ND	31.92 ± 3.38 ^A^	35.40 ± 13.13 ^A^
Rutin	1.69 ± 0.00 ^B^	1.06 ± 0.13 ^Aa^	ND	12.52 ± 5.09 ^b^
t-Cinnamic acid	0.28 ± 0.00 ^b^	ND	0.56 ± 3.97 ^a^	ND
Kaempferol	ND	0.87 ± 0.14 ^a^	ND	1.00 ± 0.20 ^a^
Quercetin	0.28 ± 0.00 ^Aa^	0.75 ± 0.20 ^Ba^	0.28 ± 1.98 ^Aa^	5.42 ± 2.29 ^Bb^
Ferulic acid	1.83 ± 0.20 ^Ba^	0.25 ± 0.00 ^A^	4.64 ± 0.60 ^b^	ND
p-Coumaric acid	0.28 ± 0.00 ^B^	0.25 ± 0.00 ^A^	ND	ND
Caffeic acid	0.70 ± 0.20 ^Aa^	1.06 ± 0.13 ^Bb^	0.28 ± 1.98 ^Aa^	0.44 ± 0.24 ^Aa^
*Resveratrol*	ND	0.58 ± 0.14 ^a^	0.98 ± 0.20 ^A^	0.75 ± 0.20 ^Aa^
Sum of phenolic compounds identified by LC-UV-MS	298.06	405.69	302.17	316.18

In the same row, superscript lowercase letters indicate differences between white and red quinoa varieties and processing methods. Superscript uppercase letters indicate differences between unfermented and fermented samples of the same quinoa color. Significant differences were computed at *p* < 0.05. WQ: unfermented white quinoa; RQ: unfermented red quinoa; FWQ: fermented white quinoa; FRQ: fermented red quinoa ND: not detectable; the concentration was traces or below the limit of detection.

## Data Availability

The original contributions presented in the study are included in the article/Supplementary Material, further inquiries can be directed to the corresponding author.

## Supplementary Materials

The following supporting information can be downloaded at https://www.mdpi.com/article/10.3390/foods13152413/s1, Figure S1: Base peak chromatogram of polyphenol mix standard solutions; Figure S2: Chromatogram for individual polyphenol standard solutions; Figure S3: Example of chromatogram of polyphenol compounds in white quinoa before fermentation; Figure S4: Example of chromatogram of polyphenol compounds in fermented white quinoa; Figure S5: Example of chromatogram of polyphenol compounds in red quinoa before fermentation; Figure S6: Example of chromatogram of polyphenol compounds in fermentedred quinoa. Examples of chromatograms for polyphenol standards in mixed solution and individually are presented along with chromatograms for samples before and after fermentation.

## Abbreviations

WQ	white quinoa, unfermented
FWQ	fermented white quinoa
RQ	red quinoa, unfermented
FRQ	fermented red quinoa
TPC	total polyphenol content
Dwb	Dried weight basis
DPPH	2,2-diphenyl-1-picrylhydrazyl
FRAP	Ferric Reducing Antioxidant Power
TAC	total antioxidant capacity
TEAC	total equivalent antioxidant capacity
Lp299v	*Lactiplantibacillus plantarum* 299v^®^
