# Supplementary material for: Effect of Short-Term Lactic Fermentation on Polyphenol Profile and Antioxidant Capacity in White and Red Quinoa Varieties
Source: Foods. 2024 Jul 30;13(15):2413. doi: 10.3390/foods13152413 (PMC11311816; doi:10.3390/foods13152413)
Supplement: Supplementary file 1 [file foods-13-02413-s001.zip › foods-3059464-supplementary.pdf]

Supplementary material

Chromatograms, LC-UV-MS determination of phenolic compounds

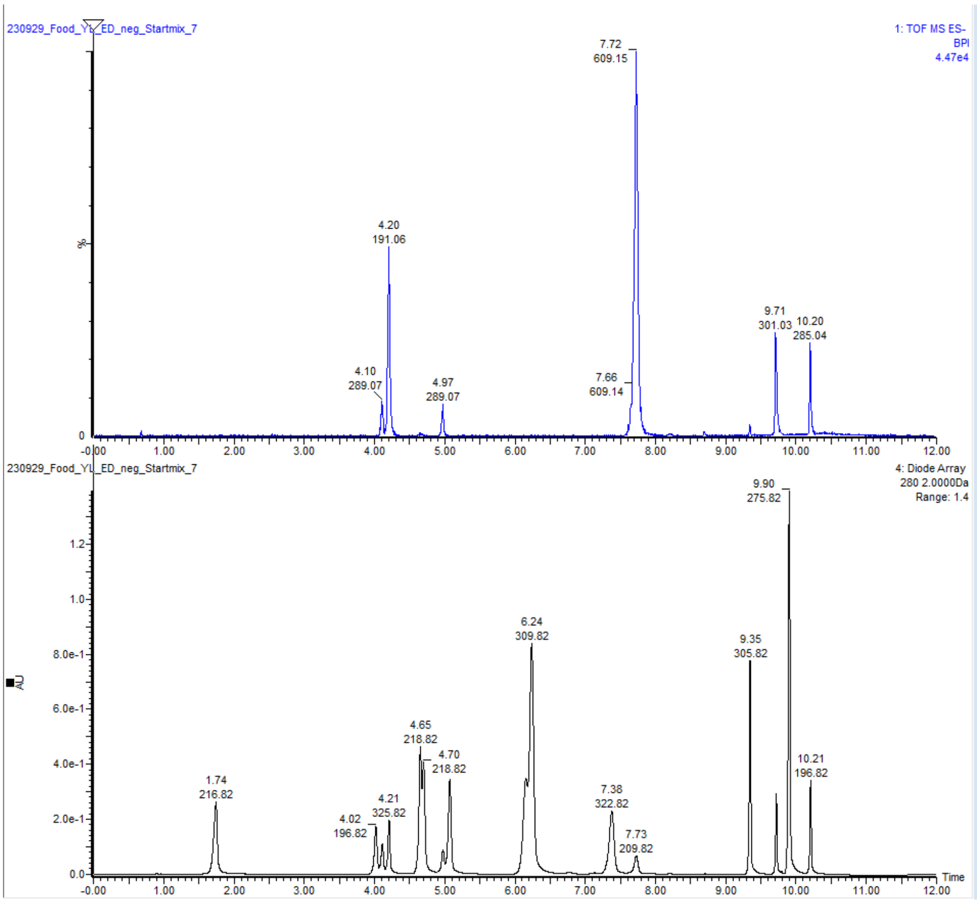

Figure S1. Base Peak chromatogram of polyphenol mix standard solutions. The peaks corespond to: Retention time Rt (min): 1.74 (gallic acid); 4.02 (4-OH Benzoic acid); 4.1 (Catechin); 4.20 (Chlorogenic acid); 4.65 (caffeic acid); 4.7 (Vanillic acid); 4.97 (Epi-catechin), 5.08 (Syringic acid), 6.16 (Vanillin); 6.24 (p-coumaric acid); 7.4 (Ferulic acid); 7.7 (rutin); 9.35 (resveratrol); 9.71 (Quercetin); 9.9 (t-cinnamic acid), 10.21 (Kaempferol).

Note that standards were also injected as individual polyphenols, example below is for vanillin and syringic acid.

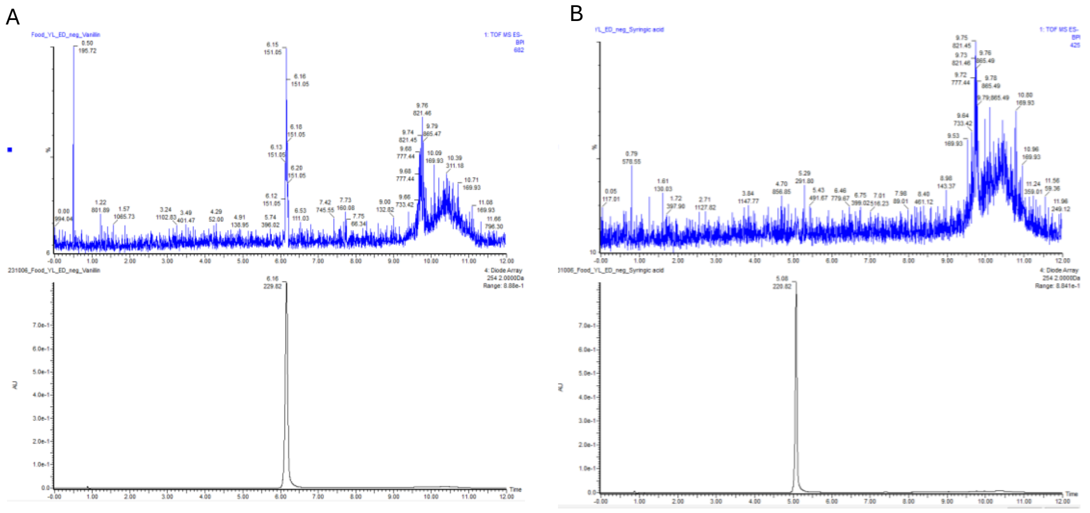

Figure S2. Chromatogram for individual polyphenol standard solutions. Examples for A Vanillin and B. Syringic acid. The peaks coresponds to: Retention time Rt (min) A. 6.16 (Vanillin) and B. 5.08 (Syringic acid),

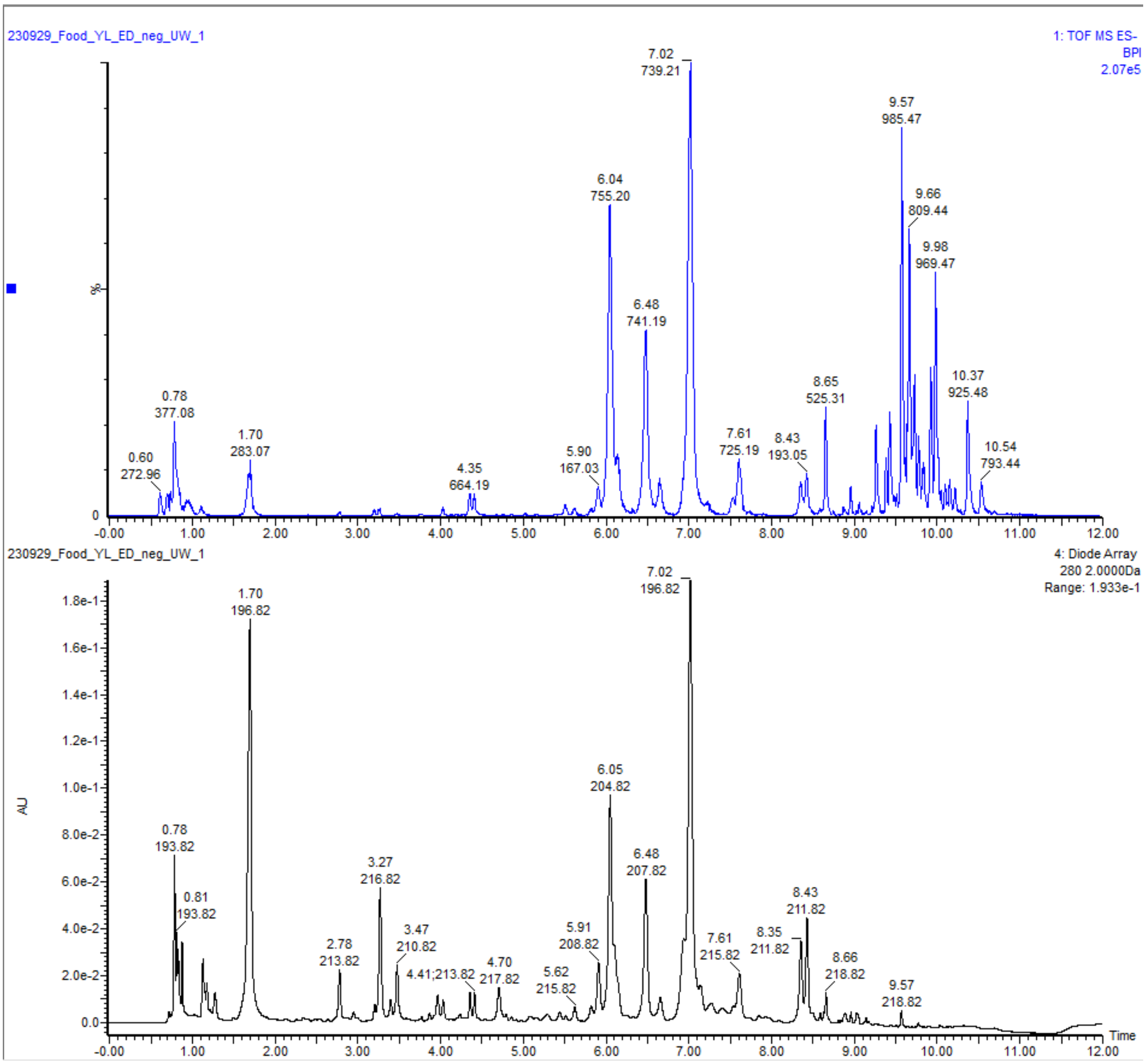

Figure S3. Example of chromatogram of polyphenol compounds in white quinoa before fermentation

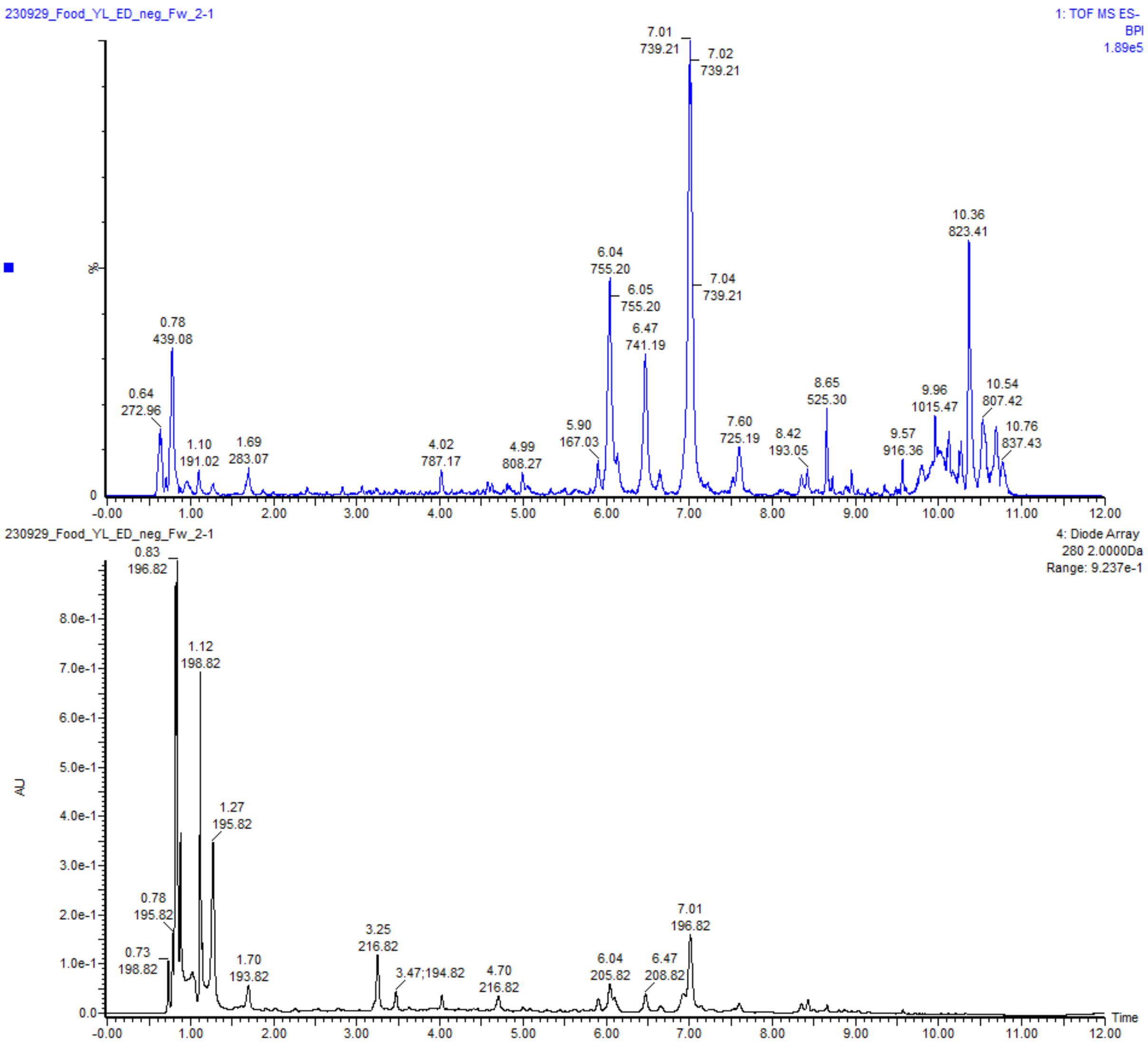

Figure S4. Example of chromatogram of polyphenol compounds in fermented white quinoa

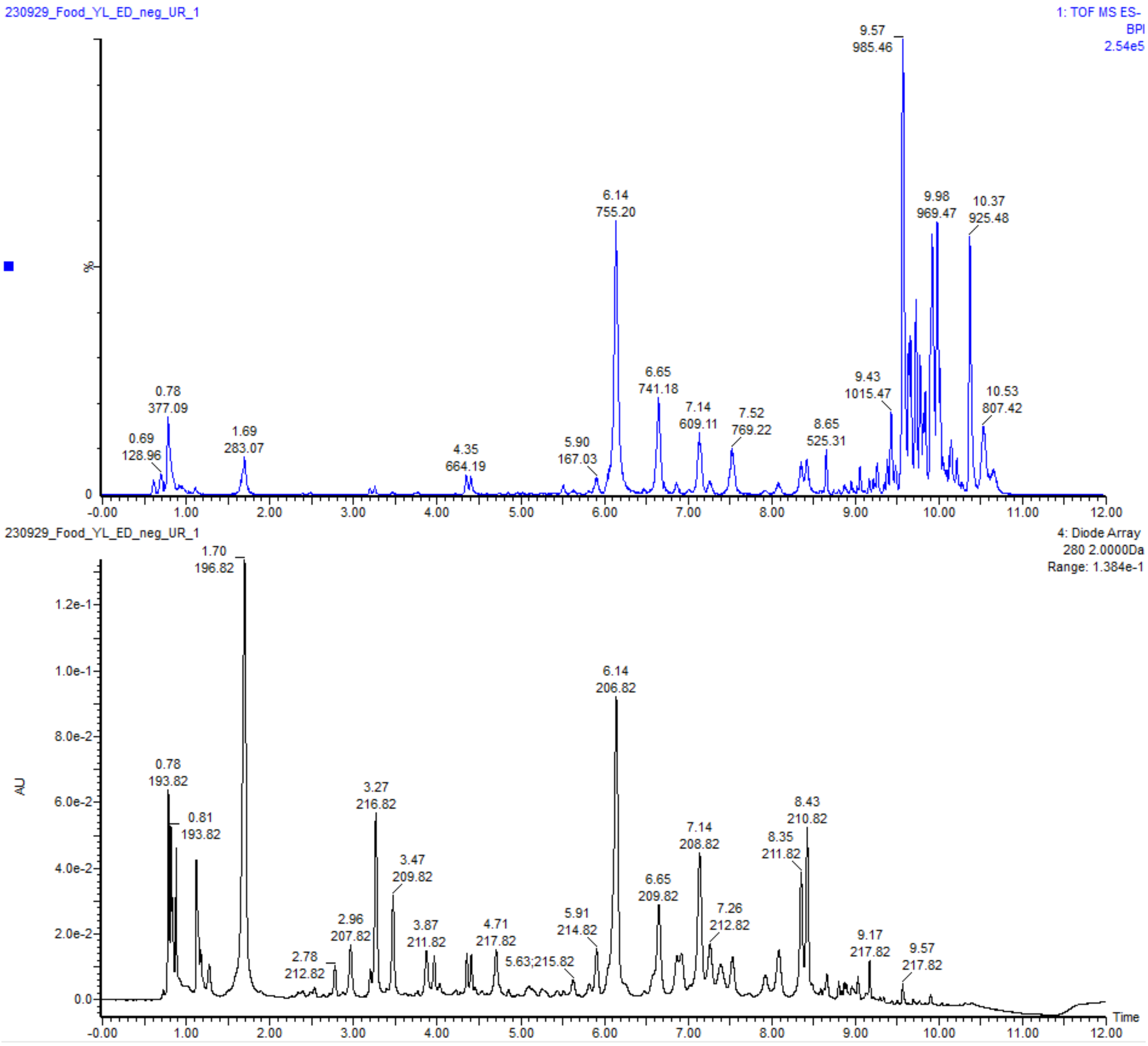

Figure S5. Example of chromatogram of polyphenol compounds in red quinoa before fermentation

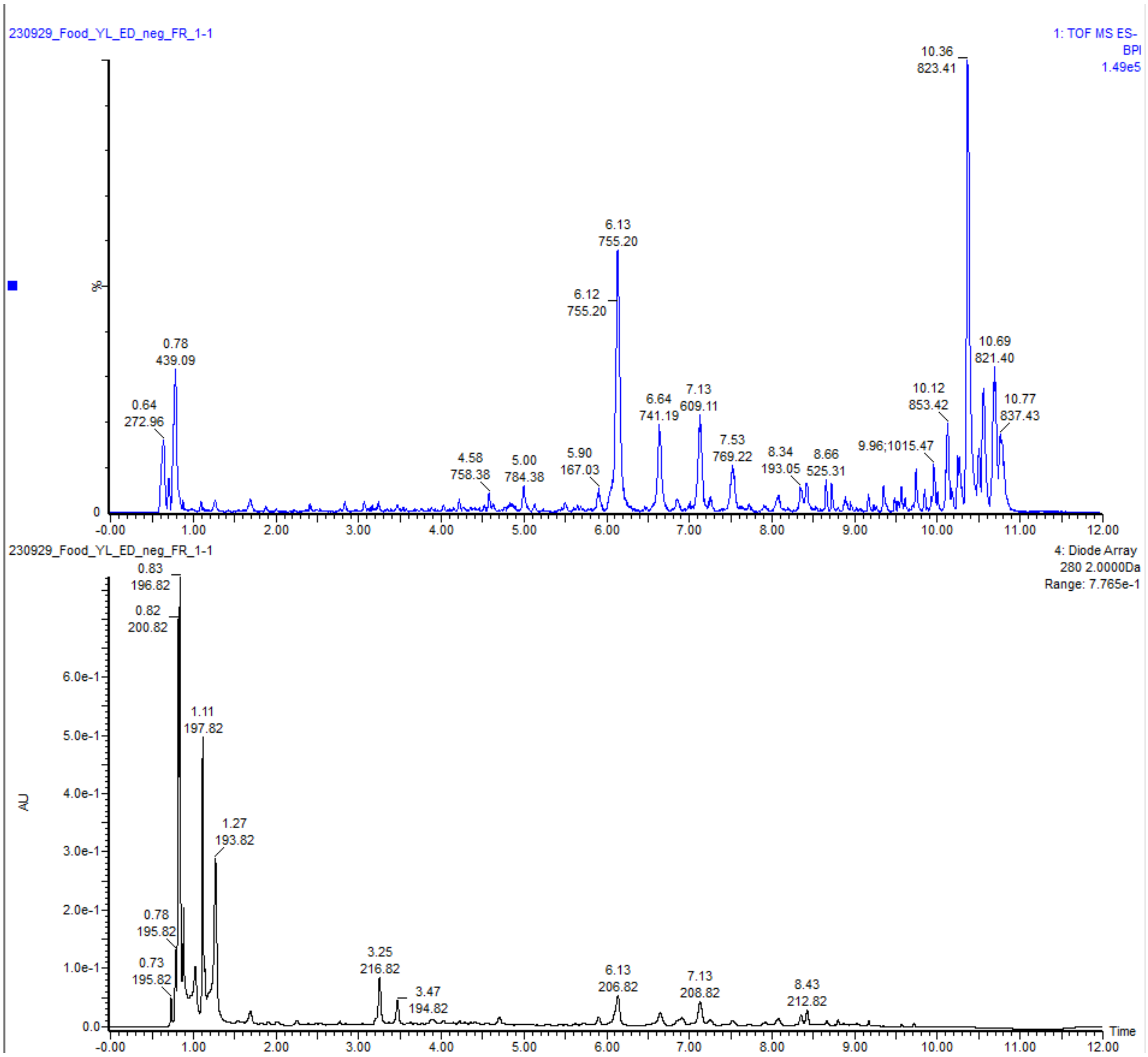

Figure S6. Example of chromatogram of polyphenol compounds in fermentedred quinoa
